# “Primum Non Nocere” in Interventional Oncology for Liver Cancer: How to Reduce the Risk for Complications?

**DOI:** 10.3390/life10090180

**Published:** 2020-09-06

**Authors:** Roberto Iezzi, Tiago Bilhim, Laura Crocetti, Bora Peynircioglu, Shraga Goldberg, Josè Ignacio Bilbao, Ahmed Sami, Okan Akhan, Paola Scalise, Felice Giuliante, Maurizio Pompili, Vincenzo Valentini, Antonio Gasbarrini, Cesare Colosimo, Riccardo Manfredi

**Affiliations:** 1 Dipartimento di Diagnostica per Immagini, Radioterapia Oncologica ed Ematologia, Fondazione Policlinico Universitario A. Gemelli-IRCCS, Università Cattolica del Sacro Cuore, L.go A. Gemelli 8, 00168 Rome, Italy; cesare.colosimo@unicatt.it (C.C.); riccardo.manfredi@unicatt.it (R.M.); 2 Nova Medical School, Interventional Radiology Unit, Saint Louis Hospital and Curry Cabral Hospital, Centro Hospitalar Universitário de Lisboa (CHULC), 1069-166 Lisbon, Portugal; tiagobilhim@hotmail.com; 3 Division of Interventional Radiology, Department of Diagnostic and Interventional Radiology and Nuclear Medicine, Cisanello University Hospital, 56126 Pisa, Italy; laura.crocetti@med.unipi.it (L.C.); scalisepl@gmail.com (P.S.); 4 Department of Radiology, Faculty of Medicine, Hacettepe University, 06100 Ankara, Turkey; borapeynir@gmail.com (B.P.); akhano@tr.net (O.A.); 5 Image-Guided Therapy and Interventional Oncology Unit, Department of Radiology, Hadassah Hebrew University Medical Center Ein Karem, 91120 Jerusalem, Israel; sgoldber@bidmc.harvard.edu; 6 Minimally Invasive Tumor Therapy Laboratory Section of Interventional Radiology, Department of Radiology Beth Israel Deaconess Medical Center/Harvard Medical School, 330 Brookline Ave Boston, Boston, MA 02215, USA; 7 Department of Radiology, Clínica Universidad de Navarra, Avenida Pio XII, no. 36, 31008 Pamplona, Spain; jibilbao@unav.es; 8 Department of Radiology, Cairo University, Cairo 12613, Egypt; asamisaeed@gmail.com; 9 Hepatobiliary Surgery Unit, Fondazione Policlinico Universitario A. Gemelli-IRCCS, Università Cattolica del Sacro Cuore, L.go A. Gemelli 8, 00168 Rome, Italy; felice.giuliante@unicatt.it; 10 Internal Medicine, Gastroenterology and Hepatology, Fondazione Policlinico Universitario A. Gemelli-IRCCS, Università Cattolica del Sacro Cuore, L.go A. Gemelli 8, 00168 Rome, Italy; maurizio.pompili@unicatt.it (M.P.); antonio.gasbarrini@unicatt.it (A.G.); 11 Gemelli Advanced Radiation Therapy Center, Fondazione Policlinico Universitario A. Gemelli-IRCCS, Università Cattolica del Sacro Cuore, L.go A. Gemelli 8, 00168 Rome, Italy; vincenzo.valentini@unicatt.it

**Keywords:** cancer, locoregional treatment, ablation, chemoembolization, radioembolization, safety, complications

## Abstract

Interventional oncology represents a relatively new clinical discipline based upon minimally invasive therapies applicable to almost every human organ and disease. Over the last several decades, rapidly evolving research developments have introduced a newer generation of treatment devices, reagents, and image-guidance systems to expand the armamentarium of interventional oncology across a wide spectrum of disease sites, offering potential cure, control, or palliative care for many types of cancer patients. Due to the widespread use of locoregional procedures, a comprehensive review of the methodologic and technical considerations to optimize patient selection with the aim of performing a safe procedure is mandatory. This article summarizes the expert discussion and report from the Mediterranean Interventional Oncology Live Congress (MIOLive 2020) held in Rome, Italy, integrating evidence-reported literature and experience-based perceptions as a means for providing guidance on prudent ways to reduce complications. The aim of the paper is to provide an updated guiding tool not only to residents and fellows but also to colleagues approaching locoregional treatments.

## 1. Introduction

The Latin aphorism “primum non nocere”, meaning “first, do no harm”, most often attributed to Hippocrates of Kos himself, is one of the principal precepts guiding all medical interventions. However, as wisely underlined by Sokol, even if a literal reading of the expression would lead clinicians to do nothing at all, a more applicable formulation should be “primum non plus nocere quam succurrere”, that is “first do no net harm”. For achieving this aim, clinicians must balance their primary obligation to benefit the patient against their obligation not to cause harm [1]. It is from this perspective that this concept must be strictly applied in interventional oncology, a relatively new clinical discipline based on minimally invasive therapies treating almost every human organ and solid cancer type. Over the last several decades, rapidly evolving research developments have introduced a newer generation of treatment devices, reagents, and image-guidance systems to expand the armamentarium of interventional oncology across a wide spectrum of disease sites (e.g., liver, lung, renal, and bone) offering potential cure, control, or palliative care for many types of cancer patients [2,3,4,5].

The adaptation of interventional radiology procedures to target and treat cancer exemplifies a new generation of promising treatment options beyond traditional surgery, chemo- and radiation therapy. However, a main current issue relates to the lack of clear indications as to the optimal therapy in some clinical situations. This mandates a comprehensive review of the methodologic and technical considerations to optimize patient selection with the aim of performing a safe procedure.

This article summarizes the expert discussion and report from the Mediterranean Interventional Oncology Live Congress (MIOLive 2020) that was held in Rome, Italy, integrating evidence-reported literature and experience-based perceptions, providing guidance as to prudent ways to achieve reducing complications, particularly for the most commonly treated organ, the liver. All attempts have been made to make the information easy to access by using a point-by-point format. Our goal is to assist not only residents and fellows who are training in interventional radiology, but also practicing colleagues who are gaining greater familiarity with percutaneous or intra-arterial treatments.

## 2. How to Reduce the Risk for Complications in Interventional Oncology

Reduced complication rates can be achieved by both applying general concepts common to all interventional procedures as well as considering several treatment-specific issues. These aspects can be grouped in four categories related to: (1) a multidisciplinary approach; (2) patient selection, (3) treatment planning, and (4) knowledge of predisposing factors for complications, as well as clinical factors affecting treatment efficacy.

### 2.1. Patient-Based Multidisciplinary Approach

The first potential opportunity to pursue the principle of minimizing complications is by maximally sharing information and decisions following a patient-based multidisciplinary approach. Multi-Disciplinary Teams (MDT) are an alliance of all medical and healthcare professionals related to a specific disease, whose approach to cancer care is guided by their willingness to agree on evidence-based clinical decisions and to coordinate the delivery of care at all stages of the process, enriched by in turn encouraging patients to take an active role in their care [6]. By means of a shared patient-centered perspective based on evidence, MDTs are intended to improve therapeutic management and treatment protocols aiming to obtain improvement in patient clinical outcomes [7]. Multidisciplinary evaluation takes into consideration the clinical specificities beyond tumor burden, such as comorbidities, compliance to treatment, general performance status, and history of the disease in order to select the best approach for the individual patient following the principles of the prescription of a treatment plan tailored as precision medicine. It has been clearly demonstrated that patients have better results in terms of treatment efficacy and overall survival or disease-free survival if single cases are preliminary discussed by MDT when compared to direct referral [8,9,10]. It is imperative to underscore that interdisciplinarity in oncology implies a synergistic application of medical therapy concepts with a focus on prolongation of survival and prevention of tumor progression. Then, locoregional therapies should be applied in order to achieve local tumor control, relief of tumor-related symptoms, and maintenance of quality of life (QoL). Furthermore, an MDT-managed treatment strategy needs to be maintained for the entire duration of a patient’s management, to allow the refinement of treatment strategies according to the response to a selected treatment and to the evaluation of the eventual need for integration of ablative and intra-arterial treatments [11,12,13].

### 2.2. Select the Right Therapeutic Option for the Right Patient at the Right Time

The second point is based on an accurate selection of the proper therapeutic option from the wide array of available modalities, based upon a thorough knowledge of all exclusion criteria and procedural risks.

#### 2.2.1. Ablation

Regarding liver tumors, ablation has a key role in the treatment of hepatocellular carcinoma (HCC) at its very early stages even as a first-line therapy replacing surgery in the case of a single tumor smaller than 2 cm; it is also an alternative to surgical resection or liver transplantation in early-stage patients with 2–3 nodules up to 3 cm, in whom the decision to perform surgery is questionable due to tumor-related factors, such as size and location, and patient-related factors, such as hepatic functional reserve, severity of portal hypertension, and extrahepatic co-morbidities [14]. For colorectal liver metastasis, the European Society for Medical Oncology (ESMO) guidelines include ablation in the toolbox of instruments for local treatment of oligometastatic disease [15]. In oligometastatic patients with unresectable liver disease, ablation may be performed in association with chemotherapy and, eventually, surgery. In patients affected by intrahepatic cholangiocarcinoma as well as neuroendocrine liver metastasis, locoregional treatments are potentially therapeutic options to cure and/or improve outcomes in unresectable patients with limited disease [16,17]. Therefore, ablation by definition can be considered a less invasive therapeutic approach, since it can be applied when other options, most of all surgery, are at high risk, as well as in unfit patients.

To increase the efficacy of ablation, new devices and techniques have been developed, such as expandable multi-tined devices, internally cooled electrodes, multipolar ablation using bipolar electrodes, microwave ablation (MWA), cryoablation, and reversible and irreversible electroporation [2]. Most of these new options try to overcome radiofrequency ablation (RFA) limitations, mainly represented by potential thermal issue of vital adjacent structures, heat sink effect if lesions are adjacent to major vessels, and tissue dependence. In detail, MWA implicates reaching fast a high temperature, thus reducing the heat sink effect, even in the absence of a reliable end point to set the amount of energy deposition. Cryoablation allows an easy monitoring of ice ball progression, decreasing the risk of thermal injuries to vital structures, even if it is supported by limited scientific evidence. Electroporation poses a limited risk of thermal injury to neighboring critical structures and is also unsensitive to the heat sink effect but it is limited by the need of general anesthesia and preliminary clinical data [18].

Furthermore, in the last years, a multimodality approach has also emerged, combining percutaneous ablation with intra-arterial procedures, extending the clinical indication and application of the percutaneous approach. In detail, based on a literature review, a combined treatment seems to be a safe and effective option in the treatment of patients with early/intermediate HCC when surgical resection is not feasible. Furthermore, this approach provides better results than RFA and transarterial chemoembolization (TACE) alone for the treatment of large HCC, defined as those exceeding 3 cm in size. It can also expand the indication for RFA to previously contraindicated “complex cases”, with increased risk of thermal ablation-related complications due to tumor location, or to “complex patients” at a high bleeding risk [19]. These treatment modalities for HCC were also applied for metastatic hepatic tumors, expanding the potential curative role of the percutaneous approach.

Moreover, RFA could also be performed alone or in combination with liver resection using a laparoscopic approach or during open surgery [18,20]. Recently, it has been observed that the combination of RFA and immunotherapy could allow achieving better outcomes in comparison to the RFA alone, in terms of improving survival rates and avoiding recurrence, for lesions larger than 3 cm in size. The association between RFA and immunomodulators induces a synergistic anticancer immune response as observed in pre-clinical studies, which seems quite promising for the future of oncological treatment [21].

#### 2.2.2. Chemoembolization

Unlike ablation that induces the precise destruction of liver tumors with limited damage to surrounding hepatic parenchyma, TACE does not allow for a precise destruction of liver tumors. Rather, TACE, even when employing super-selective techniques, allows for a degree of “shotgun” imprecision, targeting a broader area of the liver, which contains both the tumor and healthy tissue [3,22,23,24]. Thus, when considering patients eligible for TACE, a fine equilibrium between liver function and tumor volume requiring treatment must be weighed in order to treat efficiently, minimizing the potential harm to the patient. This is especially true when considering patients with chronic hepatic disease (CHD), which is frequently associated with HCC. Balancing the liver functional reserve and the non-tumoral liver volume that needs treatment is essential for patient selection and for achieving the best result in terms of local tumor control and patient’s survival benefit, avoiding unnecessary or obvious harm [3,25].

The guidelines of the European Association for the Study of the Liver–European Organisation for Research and Treatment of Cancer recommend TACE as a first-line treatment for asymptomatic patients with preserved liver function who have multinodular lesions but show no evidence of vascular invasion or extrahepatic spread (intermediate stage, or stage B in the Barcelona Clinic Liver Cancer (BCLC) staging system) [26,27]. TACE is also an appropriate palliative treatment for patients who require curative treatment but are not suitable for surgery or percutaneous ablation [28].

Chemoembolization can be performed using the conventional technique (cTACE, a two-stage treatment with a drug mixed to lipiodol followed by embolic material) or using calibrated microspheres loaded with a drug (drug-eluted microspheres (DEM)-TACE). Indications and contraindications for DEM-TACE are similar to those for c-TACE, even if DEM-TACE is considered a better option than c-TACE for patients with more advanced disease or cardiac failure and for those expected to experience severe post-embolic toxicity [29].

In the last years, new degradable beads have emerged with the possibility to overcome TACE contraindications. In detail, TACE based on degradable starch microspheres (Embocept) allows the temporary occlusion of the smaller arterial vessels, so improving the overall therapeutic effectiveness by reducing the immediate wash-out of the cytostatic agent and decreasing the risk of systemic toxicity and post-embolic syndrome. These advantages allow the safe use of this procedure also in locally advanced cases, as a second-line option in patients dismissed or ineligible for systemic options [30]. Due to a 3D cross-linking reaction, degradable beads can be mixed with a range of drugs, with indication also for secondary liver tumors and in palliative setting, also combined with systemic options, increasing drug concentration in the liver and reducing systemic toxicity and procedural adverse events [31].

#### 2.2.3. Radioembolization

Transarterial radioembolization (TARE) is a catheter-directed procedure that has be used mainly for palliation and more recently for curative purposes, with increasing frequency in the case of unresectable locally advanced primary tumors or liver-only/dominant metastatic disease. TARE is based upon the intra-arterial delivery of beta emitter Yttrium-90 (Yt-90)-embedded microspheres to achieve local therapeutic doses in the target area, with a minimal systemic effect of radiation. There are two commercially available Yt-90 preparations—glass and resin. Although both have shown to be safe and effective, they differ in terms of size, specific gravity, and activity per microsphere [4]. Holmium-166 (Ho-166) is a more recently described alternative to Yt-90 as a beta emitter. Ho-166 also emits gamma radiation, and Holmium itself is paramagnetic; these properties allow dosimetry through quantitative analysis of scintigraphic and Magnetic Resonance (MR) images [32].

TARE has shown promising results, with a safe profile; however, it is still not recommended as the standard of therapy for HCC. Nevertheless, given that TACE has its own limitations in the treatment of the subgroups of patients with large-size HCC (>5 cm) and/or portal vein neoplastic thrombosis (PVT), TARE is a reliable alternative therapy for these patients [33,34]. Indeed, the most common indication for TARE in patients with HCC is PVT. In patients with HCC, TARE can be also used as a bridge to transplantation to maintain or even reduce the size of the tumor burden while maintaining patients on the waiting list [35]. Additionally, TARE has been used to induce hypertrophy of untreated hemi-liver as an alternative to portal vein embolization prior to surgery [36,37].

Prospective randomized clinical trials (RCT) have been conducted to assess the efficacy and safety of TARE in those patients. The SARAH and SIRveNIB multi-center RCTs compared the efficacy and safety of sorafenib (a multi-kinase inhibitor which is the standard systemic therapy for advanced HCC) to those of TARE using Yt-90 resin microspheres in patients with unresectable HCC and failed to demonstrate the superiority of TARE over sorafenib as regards overall survival (OS) [38,39]. However, these studies reported significantly better tolerability and local tumor control in patients treated with TARE in comparison to those administered sorafenib. Additionally, the SARAH trial reported better QoL scores for TARE-treated patients compared to those treated with sorafenib, an important aspect of oncologic decision-making [40]. The Soramic Trial Palliative Cohort, a multi-center, multi-nation RCT that compared the efficacy and safety of a combination of sorafenib and TARE using Yt-90 resin microspheres to those of sorafenib alone in patients with unresectable HCC, reported that the combination of sorafenib and TARE did not demonstrate a significant improvement in OS [41]. Although the safety and toxicity profiles were similar between groups, subgroup analyses suggested a possible clinical benefit of TARE plus sorafenib for non-cirrhotic patients, patients with non-alcoholic etiology of liver disease, and younger patients.

Looking at metastatic liver disease, SIRFLOX was a multi-center RCT aimed to assess the efficacy and safety of adding TARE with Y-90 resin microspheres to chemotherapy based on fluorouracil, leucovorin, and oxaliplatin (FOLFOX) for the treatment of patients with liver-only/dominant metastatic colorectal cancer (mCRC) as a first-line treatment [41]. Although the study showed no benefit of adding TARE to FOLFOX for progression free survival (PFS) at any site, the combination of TARE and FOLFOX improved median PFS in the liver by 7.9 months, with a 31% risk reduction for disease progression. Moreover, the REsect study demonstrated that adding TARE to chemotherapy significantly increased the number of patients with colorectal metastases who became amenable to resection at follow-up [42,43]. TARE has also shown to be effective and safe, with promising results in patients with unresectable, chemorefractory intrahepatic cholangiocarcinoma and metastatic neuroendocrine and breast cancer metastasis [44,45,46,47].

### 2.3. Perform Proper Treatment Planning and Define Correct Procedural Technique

#### 2.3.1. Ablation

When performing an ablative treatment, selection of proper imaging guidance is mandatory. The target to treat should be visualized under the guidance of ultrasound (US) and/or computed tomography (CT) based upon location and conspicuity, and treatment can also be performed with videolaparoscopic (VSL) and/or robotic approaches. In addition, guidance and monitoring can be improved by means of stereotactic guidance systems, which represent a useful aid to improve accuracy especially in difficult locations and/or when complex multiple treatments are needed, combining the possibility of complete ablation of the tumor with the preservation of delicate surrounding structures and minimizing destruction of healthy tissue [2].

When considering potential complications, technical aspects, usually related to the target tumor location, must also be evaluated. As an example, direct applicator insertion into superficial nodules with a subcapsular location or invasive tumoral pattern can cause needle track seeding in up to 2.5% of patients (range 0–12.5%) if ablation followed diagnostic biopsy [48]. Thus, direct puncture should be avoided, and transhepatic paths or a “no touch of the tumor” technique should be used to avoid tumoral seeding [2]. Peribiliary nodules are another difficult challenge, especially when adjacent to the main biliary ducts. To protect the biliary system, a modulated ablation technique and/or duct cooling can be performed. Cooling can be accomplished intraoperatively, percutaneously, or by an endoscopic nasobiliary drainage tube [49]. Nodules in close proximity to gut and gallbladder can be approached with hydrodissection or gas dissection in order to separate the gallbladder from the heated zone and to reduce complications. Solutions of non-conductive 5% dextrose in water (5% DW) for RFA and of 0.9% NaCl for MWA should be used and can be mixed with an iodinated contrast medium to improve visualization [50]. Self-limited morbidity is noted in most patients after ablation of nodules adjacent to the gallbladder, most often attributed to a mild iatrogenic cholecystitis [51]. To reduce this complication, the ablation zone can be reconfigured by offsetting the applicator from the center of the tumor [52]. A combined angio/percutaneous approach is advisable in case of centrally located intermediate-size tumors due to the high probability of transverse arterial vessels reaching the tumor [53]. In the last years, to improve the placement of the ablation needle in the tumor and an adequate treatment planning, a stereotactic image guidance has been proposed, allowing a better coverage of a tumor with necrosis, with an accurate safety margin of at least 5 to 10 mm, reducing the risk of residual disease or local tumor progression and influencing survival prognosis [54]. Stereotactic ablation could also allow the treatment of complex lesions in difficult-to-reach location with low complication rate, also facilitating complicated access routes [55].

#### 2.3.2. Intra-Arterial Treatments

For intra-arterial treatments such as TACE and TARE, the use of C-arm cone-beam computed tomography (CBCT) is becoming mandatory to overcome the substantial limitations of two-dimensional angiography alone [56]. Specifically, CBCT can help interventional radiologists to visualize small tumors and tumor-feeding arteries, identify occult lesions and the 3D configuration of tortuous hepatic arteries. Furthermore, it can suggest the presence of extrahepatic collateral arteries supplying the tumors, minimize nontarget embolization, and confirm and reassure the operator of the completeness of the administered treatment [57,58,59,60].

New generations of microcatheters have also been developed, with the goal of allowing not only a better treatment delivery (i.e., smaller catheter size, preformed tip shape, steerable tip) but also pressure dynamic alterations which are useful to maximize treatment delivery to the tumor (in the case of low-vascular-capacitance tumors) and exposure to the targeted volume, reducing complications and non-target embolization (antireflux microcatheters) [61,62,63]. These devices can be based upon different mechanisms. An example is the microballoon microcatheter, which allows a flow redistribution and a reduction of non-target embolization, creating a fluid barrier with a unique filter tip or utilizing an expanded tip that collapses under pressure of forward-flowing blood, while also expanding to act as a valve to prevent backflow during the infusion of therapeutic agents.

##### Intra-Arterial: Chemoembolization

Although interventional radiologists are becoming ever more comfortable with superselective catheterization with microcatheters, and complications from non-target embolization are becoming less problematic, the latter still need to be considered. Avoiding selective contrast administration using high-pressure-pump injections after embolization or eliminating additional “control” angiography near the tumor target can help minimize non-target embolization due to reflux of the embolic material previously delivered. Careful hand injections or control angiography with power injections from a different catheter at a more proximal location are other options. When performing angiography before TACE, arterio-portal or arterio-hepatic venous fistulas can be seen and, when recognized, should prompt cessation of the procedure, as they can lead to biliary and liver necrosis (arterio-portal fistulas) or systemic distribution of the embolic material (arterio-hepatic vein fistulas) [25]. Another aspect to be considered when performing liver TACE is the dosage of the drug used, as high amounts of the injected drug can increase the risk of bile duct toxicity (ischemic biliopathy), leading to biloma formation, liver infarction, and abscess [3,25,64,65,66]. Likewise, it is prudent to adjust the dose of the drug to each of the currently available drug-eluting microspheres, as they can have different drug elution and release properties with respect to the liver [67].

It is also mandatory to evaluate the tumor volume and location. In order to avoid liver insufficiency from overtreatment, many physicians use the absolute upper treatment cutoff of >50% of tumor burden with respect to liver volume, while less conservative practitioners use a cutoff of >75% of liver replacement by a tumor. Moreover, large tumors present a higher risk of portal invasion or thrombosis and, thus, of liver infarction. They also frequently may reach the liver capsule, causing an increased risk of rupture and bleeding into the peritoneum. Furthermore, large tumors frequently also need to be treated with higher volumes of embolics and higher dosages of chemotherapy, and this inevitably rises the risk of hepatic failure and severe post-embolization syndrome (PES) [68,69]. Additionally, when considering location, tumors adjacent to the gallbladder may be vascularized by arterial branches of the cystic artery, or reflux of embolic material may reach the gallbladder, leading to cholecystitis. Exophytic and subcapsular tumors may recruit extra-hepatic, systemic arterial feeders, with a higher potential for extra-hepatic non-target embolization. In these situations, bland embolization may be considered instead of TACE [3].

##### Intra-Arterial: Radioembolization

Careful pretreatment planning and dosimetry play a key role in the avoidance of TARE-related complications. All patients should undergo pretreatment clinical, laboratory, and diagnostic imaging evaluation. Pretreatment screening angiography needs to be performed to assess the hepatic-mesenteric vasculature and to administer Technetium-99m macroaggregated albumin (Tc-99m MAA), which is considered to be a trial surrogate to the Yt-90, into the hepatic artery branch intended for TARE. After Tc-99m MAA administration, lung shunt and gastrointestinal leakage can be evaluated by whole-body scintigraphy. Lung shunting of 20%, estimated lung dose > 30 Gy for single session and >50 Gy for multiple sessions and uncontrollable gastrointestinal leakage are contraindication for TARE, although most potential “off-target” gastrointestinal deposition is manageable via embolization of the culprit artery (gastroduodenal, right gastric, cystic artery, etc.) or positioning of the catheter beyond the origin of these arteries [4]. Very high activities of Tc-99m MAA could lead to overtreatment in certain areas, also endangering the surrounding normal liver parenchyma. To reduce the radioactivity dose to healthy liver, a partition method using lung, healthy liver, and the tumor as separate dosimetric compartments offers a more personalized dosimetry than an empiric method based only upon the percentage of tumor involvement [70].

### 2.4. Know Treatment-Related Potential Complications and Clinical Factors Affecting Treatment Efficacy

#### 2.4.1. Ablation

Potential complications requiring medical management must be considered. They include mostly bleeding complications since, unlike surgeons, interventional radiologists usually do not have direct control of hemostasis. In recent consensus guidelines, despite its thermal coagulative properties, ablation is considered a procedure with a high risk of causing bleeding, and thus evaluation of prothrombin time (PT), International Normalised Ratio (INR), and platelet count before starting the procedure is routinely recommended. The thresholds for correction are: for INR, corrected if outside the range of 1.5–1.8; for platelet count, corrected if <50 × 10^9^/L [71]. In patients with liver cirrhosis, abnormal screening coagulation test results do not correlate with bleeding risk because of the physiology of rebalanced hemostasis, resulting in a decreased tendency to bleed than expected. In addition, splenomegaly also plays a central role in thrombocytopenia, and hyperfibrinolysis is emerging as a cause of bleeding. Therefore, transfusion strategies for the management of patients with chronic liver disease have been recently revised, though they are currently based on expert opinion rather than on formal empiric studies. The suggested laboratory thresholds for procedures with a high risk of bleeding in patients with chronic liver disease are: INR correction to <2.5; platelet transfusion if platelet count is <30 × 10^9^/L; fibrinogen > 100 mg/dL (with one dose of cryoprecipitate if <100 mg/dL) [71].

Another dreaded potential complication is infection. Ablation is classified as a “clean-contaminated” procedure, that is, a procedure performed without active inflammation that does not compromise sterility, with limited risks, mainly due to cutaneous bacteria [72]. In these low-risk patients, the large amount of necrotic material created during ablation poses a risk for bacterial seeding during percutaneous access, and the use of a single antibiotic agent targeted to the skin flora (i.e., cefazolin) is deemed most reasonable [71]. On the other hand, patients with a history of biliary colonization as a result of an incompetent sphincter of Oddi or biliary–enteric anastomosis undergoing liver tumor ablation are at substantially higher risk for the development of an abscess. In these patients, ablation should be considered a contaminated procedure, and specific prophylaxis is recommended (i.e., oral levofloxacin 500 mg/d and oral metronidazole 500 mg twice daily, beginning 2 days before and continuing for 14 days after ablation; bowel preparation with neomycin 1 g and erythromycin base 1 g orally t.i.d. on the day before ablation) [72].

#### 2.4.2. Intra-Arterial Treatments

The potential adverse events after intra-arterial treatments are most often strictly related to liver function. Specifically, most guidelines use the Child–Pugh score of >B7 and bilirubin (BrB) values > 2 mg/dL as relative contraindications for chemo/radioembolization, with some arguing that BrB values between 2 and 3 mg/dL are safe for TACE if superselective tumor embolization is achievable [3,14]. Furthermore, TACE with degradable beads can be a solution for selected advanced clinical patients ineligible for standard TACE [30] due to a poor underlying liver function.

Like ablation, biliary abnormalities such as obstruction, biliary–enteric anastomosis, or indwelling biliary stents lead to a marked increase in the risk of biloma formation, abscess, and cholangitis after liver intra-arterial treatment, potentially leading to death. Thus, the real risk–benefit profile for intra-arterial treatment needs to be discussed, and potential complications reviewed when discussing for patients’ consent. If TACE is still considered the most reasonable option, in these situations, prophylactic coverage with antibiotics is mandatory over the week following treatment (using piperaciline/tazobactam, 4 g/0.5 g, i.v. every 8 h or levofloxacin, 500 mg qd p.o. plus metronidazol 400 mg, b.i.d. p.o. for 5–7 days) [3].

By contrast, TARE has minimal or no embolic effect, as non-radioactive individual carrier microspheres prompt virtually no inflammatory reaction in the embolization zone [73]. Thus, though TARE has its own radiation-induced complications, PES is less of an issue than for TACE, which has an apparent embolic effect. The most feared TARE-related complications are radioembolization-induced liver disease (REILD) and pneumonitis, which are fortunately rare. Radiation cholecystitis, gastritis, duodenitis, and pancreatitis can also be seen and are related to offtarget radioembolization. Although REILD mostly occurs 4–8 weeks after radiation therapy, it can present as early as two weeks or as late as seven months. Its incidence ranges between 0 and 4%. Baseline abnormal hepatic function and activity applied to liver have an impact on the risk of developing REILD [74].

## 3. Conclusions

Interventional oncology is playing a pivotal emerging role in the management of primary and secondary hepatic tumors. Treatments need to be both effective and safe, with a mandatory aim to reduce the risk for complications. Factors that contribute to this aim include an adequate multidisciplinary approach, correct indication, accurate treatment planning, and comprehensive knowledge of complications as well as of clinical factors affecting treatment efficacy. A thorough knowledge of indications, complications, imaging-guidance tools, and treatment modalities is needed, as well as of alternative approaches. Patients should be asked to take an active part in their care process, and their approval of an updated informed consent after discussion of the therapeutic plan is essential.

## References

[1] Sokol D.K. (2013). “First do no harm” revisited. BMJ.

[2] Crocetti L., Iezzi R., Goldberg S.N., Bilbao J.I., Sami A., Akhan O., Giuliante F., Pompili M., Malagari K., Valentini V. (2018). The ten commandments of liver ablation: Expert discussion and report from Mediterranean Interventional Oncology (MIOLive) congress 2017. Eur. Rev. Med. Pharmacol. Sci..

[3] Malagari K., Iezzi R., Goldberg S.N., Bilbao J.I., Sami A., Akhan O., Giuliante F., Pompili M., Crocetti L., Valentini V. (2018). The ten commandments of chemoembolization: Expert discussion and report from Mediterranean Interventional Oncology (MIOLive) congress 2017. Eur. Rev. Med. Pharmacol. Sci..

[4] Bilbao J.I., Iezzi R., Goldberg S.N., Crocetti L., Sami A., Akhan O., Giuliante F., Pompili M., Malagari K., Valentini V. (2017). The ten commandments of hepatic radioembolization: Expert discussion and report from Mediterranean Interventional Oncology (MIOLive) congress 2017. Eur. Rev. Med. Pharmacol. Sci..

[5] Tsetis D., Uberoi R., Fanelli F., Roberston I., Krokidis M., Van Delden O., Radeleff B., Müller-Hülsbeck S., Szerbo-Trojanowska M., Lee M. (2016). The Provision of Interventional Radiology Services in Europe: CIRSE Recommendations. Cardiovasc. Interv. Radiol..

[6] Borras J.M., Albreht T., Audisio R., Briers E., Casali P., Esperou H., Grube B., Hamoir M., Henning G., Kelly J. (2014). Policy statement on multidisciplinary cancer care. Eur. J. Cancer.

[7] Prades J., Remue E., Van Hoof E., Borras J.M. (2015). Is it worth reorganising cancer services on the basis of multidisciplinary teams (MDTs)? A systematic review of the objectives and organisation of MDTs and their impact on patient outcomes. Health Policy.

[8] MacDermid E., Hooton G., MacDonald M., McKay G., Grose D., Mohammed N., Porteous C. (2009). Improving patient survival with the colorectal cancer multi-disciplinary team. Colorectal Dis..

[9] Du C.Z., Li J., Cai Y., Sun Y.S., Xue W.C., Gu J. (2011). Effect of multidisciplinary team treatment on outcomes of patients with gastrointestinal malignancy. World J. Gastroenterol..

[10] Lordan J.T., Karanjia N.D., Quiney N., Fawcett W.J., Worthington T.R. (2009). A 10-year study of outcome following hepatic resection for colorectal liver metastases—The effect of evaluation in a multidisciplinary team setting. Eur. J. Surg. Oncol..

[11] Weledji E.P. (2017). Centralization of liver Cancer surgery and impact on multidisciplinary teams working on stage IV colorectal Cancer. Oncol. Rev..

[12] Adam R., De Gramont A., Figueras J., Kokudo N., Kunstlinger F., Loyer E., Poston G., Rougier P., Rubbia-Brandt L., Sobrero A. (2015). Påhlman L of the EGOSLIM (Expert Group on OncoSurgery management of LIver Metastases) group. Managing synchronous liver metastases from colorectal cancer: A multidisciplinary international consensus. Cancer Treat. Rev..

[13] Oxenberg J., Papenfuss W., Esemuede I., Attwood K., Simunovic M., Kuvshinoff B., Francescutti V. (2015). Multidisciplinary cancer conferences for gastrointestinal malignancies result in measureable treatment changes: A prospective study of 149 consecutive patients. Ann. Surg. Oncol..

[14] European Association for the Study of the Liver (2018). EASL Clinical Practice Guidelines: Management of hepatocellular carcinoma. J. Hepatol..

[15] Van Cutsem E., Cervantes A., Adam R., Sobrero A., Van Krieken J.H., Aderka D., Aranda Aguilar E., Bardelli A., Benson A., Bodoky G. (2016). ESMO consensus guidelines for the management of patients with metastatic colorectal cancer. Ann. Oncol..

[16] Bridgewater J., Galle P.R., Khan S.A., Llovet J.M., Park J.W., Patel T., Pawlik T.M., Gores G.J. (2014). Guidelines for the diagnosis and management of intrahepatic cholangiocarcinoma. J. Hepatol..

[17] Pavel M., O’Toole D., Costa F., Capdevila J., Gross D., Kianmanesh R., Krenning E., Knigge U., Salazar R., Pape U.F. (2016). Vienna Consensus Conference participants. ENETS Consensus Guidelines Update for the Management of Distant Metastatic Disease of Intestinal, Pancreatic, Bronchial Neuroendocrine Neoplasms (NEN) and NEN of Unknown Primary Site. Neuroendocrinology.

[18] Nault J.C., Sutter O., Nahon P., Ganne-Carrié P., Séror O. (2018). Percutaneous treatment of hepatocellular carcinoma: State of the art and innovations. J. Hepatol..

[19] Iezzi R., Pompili M., Posa A., Coppola G., Gasbarrini A., Bonomo L. (2016). Combined locoregional treatment of patients with hepatocellular carcinoma: State of the art. World J. Gastroenterol..

[20] Cillo U., Vitale A., Dupuis D., Corso S., Neri D., D’Amico F., Gringeri E., Farinati F., Vincenzi V., Zanus G. (2013). Laparoscopic ablation of hepatocellular carcinoma in cirrhotic patients unsuitable for liver resection or percutaneous treatment: A cohort study. PLoS ONE.

[21] Da Costa A.C., Sodergren M., Jayant K., Cruz F.S., Spalding D., Pai M., Habib N. (2020). Radiofrequency combined with immunomodulation for hepatocellular carcinoma: State of the art and innovations. World J. Gastroenterol..

[22] Habib A., Desai K., Hickey R., Thornburg B., Lewandowski R., Salem R. (2015). Transarterial approaches to primary and secondary hepatic malignancies. Nat. Rev. Clin. Oncol..

[23] Miura J.T., Gamblin T.C. (2015). Transarterial chemoembolization for primary liver malignancies and colorectal liver metastasis. Surg. Oncol. Clin. N. Am..

[24] Lewandowski R.J., Geschwind J.F., Liapi E., Salem R. (2011). Transcatheter intraarterial therapies: Rationale and overview. Radiology.

[25] Gaba R.C., Lokken R.P., Hickey R.M., Lipnik A.J., Lewandowski R.J., Salem R., Brown D.B., Walker T.G., Silberzweig J.E., Baerlocher M.O. (2017). Quality Improvement Guidelines for Transarterial Chemoembolization and Embolization of Hepatic Malignancy. J. Vasc. Interv. Radiol..

[26] Nouri Y.M., Kim J.H., Yoon H.K., Ko H.K., Shin H.K., Gwon D. (2019). Update on Transarterial Chemoembolization with Drug-Eluting Microspheres for Hepatocellular Carcinoma. Korean J. Radiol..

[27] Kim J.H., Shim J.H., Lee H.C., Sung K.B., Ko H.K., Ko G.Y., Gwon D.I., Kim J.W., Lim Y.S., Park S.H. (2017). New intermediate-stage subclassification for patients with hepatocellular carcinoma treated with transarterial chemoembolization. Liver Int..

[28] Han K., Kim J.H. (2015). Transarterial chemoembolization in hepatocellular carcinoma treatment: Barcelona clinic liver cancer staging system. World J. Gastroenterol..

[29] Nouri Y.M., Kim J.H., Yoon H.K., Ko H.K., Shin J.H., Gwon D.I. (2016). Drug-eluting bead transarterial chemoembolization as bridge therapy for hepatocellular carcinoma before living-donor liver transplantation. Transplant. Proc..

[30] Iezzi R., Pompili M., Rinninella E., Annicchiarico E., Garcovich M., Cerrito L., Ponziani F., De Gaetano A., Siciliano M., Basso M. (2019). HepatoCatt Study Group. TACE with degradable starch microspheres (DSM-TACE) as second-line treatment in HCC patients dismissing or ineligible for sorafenib. Eur. Radiol..

[31] Schicho A., Pereira P.L., Michalik K., Beyer L.P., Stroszczynski C., Wiggermann P. (2018). Safety and efficacy of transarterial chemoembolization with degradable starch microspheres (DSM-TACE) in the treatment of secondary liver malignancies. Onco Targets Ther..

[32] Smits M.L.J., Nijsen J.F.W., Van den Bosch M.A.A.J., Lam M.G.E.H., Vente M.A.D., Huijbregts J.E., Schip A.D.V., Elschot M., Bult W., De Jong H.W.A.M. (2010). Holmium-166 radioembolization for the treatment of patients with liver metastases: Design of the phase I HEPAR trial. J. Exp. Clin. Cancer Res..

[33] Kulik L.M., Carr B.I., Mulcahy M.F., Lewandowski R.J., Atassi B., Ryu R.K., Sato K.T., Benson A., Nemcek A.A., Gates V.L. (2008). Safety and efficacy of 90Y radiotherapy for hepatocellular carcinoma with and without portal vein thrombosis. Hepatol. Jan..

[34] Golfieri R., Renzulli M., Mosconi C., Forlani L., Giampalma E., Piscaglia F., Trevisani F., Bolondi L., Bologna Liver Oncology Group (2013). Hepatocellular carcinoma responding to superselective transarterial chemoembolization: An issue of nodule dimension?. J. Vasc. Interv. Radiol. JVIR.

[35] Tohme S., Sukato D., Chen H., Amesur N., Zajko A.B., Humar A., Geller D.A., Marsh J.W., Tsung A. (2013). Yttrium-90 radioembolization as a bridge to liver transplantation: A single-institution experience. J. Vasc. Interv. Radiol. JVIR.

[36] Edeline J., Lenoir L., Boudjema K., Rolland Y., Boulic A., Le Du F., Pracht M., Raoul J., Clément B., Garin E. (2013). Volumetric changes after (90)y radioembolization for hepatocellular carcinoma in cirrhosis: An option to portal vein embolization in a preoperative setting?. Ann. Surg. Oncol..

[37] Fernández-Ros N., Silva N., Bilbao J.I., Iñarrairaegui M., Benito A., D’Avola D., Rodriguez M., Rotellar F., Pardo F., Sangro B. (2014). Partial liver volume radioembolization induces hypertrophy in the spared hemiliver and no major signs of portal hypertension. HPB Off. J. Int. Hepatol. Pancreato Biliary Assoc..

[38] Vilgrain V., Pereira H., Assenat E., Guiu B., Ilonca A.D., Pageaux G., Sibert A., Bouattour M., Lebtahi R., Allaham W. (2017). Efficacy and safety of selective internal radiotherapy with yttrium-90 resin microspheres compared with sorafenib in locally advanced and inoperable hepatocellular carcinoma (SARAH): An open-label randomised controlled phase 3 trial. Lancet Oncol..

[39] Chow P.K.H., Gandhi M., Tan S., Khin M.W., Khasbazar A., Ong J., Choo S.P., Cheow P.C., Chotipanich C., Lim K. (2018). SIRveNIB: Selective Internal Radiation Therapy Versus Sorafenib in Asia-Pacific Patients with Hepatocellular Carcinoma. J. Clin. Oncol. Off. J. Am. Soc. Clin. Oncol..

[40] Das A., Gabr A., O’Brian D.P., Riaz A., Desai K., Thornburg B., Kallini J.R., Mouli S., Lewandowski R.J., Salem R. (2019). Contemporary Systematic Review of Health-Related Quality of Life Outcomes in Locoregional Therapies for Hepatocellular Carcinoma. J. Vasc. Interv. Radiol. JVIR..

[41] Ricke J., Klümpen H.J., Amthauer H., Bargellini I., Bartenstein P., De Toni E.N., Gasbarrini A., Pech M., Peck-Radosavljevic M., Popovič P. (2019). Impact of combined selective internal radiation therapy and sorafenib on survival in advanced hepatocellular carcinoma. J. Hepatol..

[42] Garlipp B., Gibbs P., Van Hazel G.A., Jeyarajah R., Martin R.C.G., Bruns C.J., Lang H., Manas D.M., Ettorre G.M., Pardo F. (2017). REsect: Blinded assessment of amenability to potentially curative treatment of previously unresectable colorectal cancer liver metastases (CRC LM) after chemotherapy ± RadioEmbolization (SIRT) in the randomized SIRFLOX trial. J. Clin. Oncol..

[43] Wasan H.S., Gibbs P., Sharma N.K., Taieb J., Heinemann V., Ricke J., Peeters M., Findlay M., Weaver A., Mills J. (2017). First-line selective internal radiotherapy plus chemotherapy versus chemotherapy alone in patients with liver metastases from colorectal cancer (FOXFIRE, SIRFLOX, and FOXFIRE-Global): A combined analysis of three multicentre, randomised, phase 3 trials. Lancet Oncol..

[44] Padia S.A. (2019). Y90 Clinical Data Update: Cholangiocarcinoma, Neuroendocrine Tumor, Melanoma, and Breast Cancer Metastatic Disease. Tech. Vasc. Interv. Radiol..

[45] Gordon A.C., Gradishar W.J., Kaklamani V.G., Thuluvath A.J., Ryu R.K., Sato K.T., Gates V.L., Salem R., Lewandowski R.J. (2014). Yttrium-90 Radioembolization Stops Progression of Targeted Breast Cancer Liver Metastases after Failed Chemotherapy. J. Vasc. Interv. Radiol..

[46] Jia Z., Wang W. (2018). Yttrium-90 radioembolization for unresectable metastatic neuroendocrine liver tumor: A systematic review. Eur. J. Radiol..

[47] Filippi L., Schillaci O., Cianni R., Bagni O. (2018). Yttrium-90 resin microspheres and their use in the treatment of intrahepatic cholangiocarcinoma. Future Oncol..

[48] Stigliano R., Marelli L., Yu D., Davies N., Patch D., Burroughs A.-K. (2007). Seeding following percutaneous diagnostic and therapeutic approaches for hepatocellular carcinoma. What is the risk and the outcome?. Cancer Treat. Rev..

[49] Elias M., Sideris L., Pocard M., Dromain C., De Baere T. (2004). Intraductal cooling of the main bile ducts during radiofrequency ablation prevents biliary stenosis1 1No competing interests declared. J. Am. Coll. Surg..

[50] Kondo Y., Yoshida H., Shiina S., Tateishi R., Teratani T., Omata M. (2006). Artificial ascites technique for percutaneous radiofrequency ablation of liver cancer adjacent to the gastrointestinal tract. BJS.

[51] Chopra S., Dodd G.D., Chanin M.P., Chintapalli K.N. (2003). Radiofrequency Ablation of Hepatic Tumors Adjacent to the Gallbladder: Feasibility and Safety. Am. J. Roentgenol..

[52] Choi I.Y., Kim P.-N., Lee S.G., Won H.J., Shin Y.M. (2017). Efficacy and Safety of Radiofrequency Ablation for Focal Hepatic Lesions Adjacent to Gallbladder: Reconfiguration of the Ablation Zone through Probe Relocation and Ablation Time Reduction. J. Vasc. Interv. Radiol..

[53] Iezzi R., Pompili M., Posa A., Carchesio F., Siciliano M., Annicchiarico B.E., Agnes S., Giuliante F., Garcovich M., Cerrito L. (2019). Interventional oncology treatments for unresectable early stage HCC in patients with a high risk for intraprocedural bleeding: Is a single-step combined therapy safe and feasible?. Eur. J. Radiol..

[54] Bale R., Schullian P., Eberle G., Putzer D., Zoller H., Schneeberger S., Manzl C., Moser P., Oberhuber G. (2019). Stereotactic Radiofrequency Ablation of Hepatocellular Carcinoma: A Histopathological Study in Explanted Livers. Hepatology.

[55] Schullian P., Putzer D., Laimer G., Levy E., Bale R. (2019). Feasibility, safety, and long-term efficacy of stereotactic radiofrequency ablation for tumors adjacent to the diaphragm in the hepatic dome: A case-control study. Eur. Radiol..

[56] Kim H.-C. (2015). Role of C-Arm Cone-Beam CT in Chemoembolization for Hepatocellular Carcinoma. Korean J. Radiol..

[57] Bapst B., Lagadec M., Bréguet R., Vilgrain V., Ronot M. (2015). Cone Beam Computed Tomography (CBCT) in the Field of Interventional Oncology of the Liver. Cardiovasc. Interv. Radiol..

[58] Yao X., Yan N., Jiang X., Li X., Zeng H., Liu D., Li H. (2018). Dual-phase Cone-beam CT-based Navigation Imaging Significantly Enhances Tumor Detectability and Aids Superselective Transarterial Chemoembolization of Liver Cancer. Acad. Radiol..

[59] Pung L., Ahmad M., Mueller K., Rosenberg J., Stave C.D., Hwang G.L., Shah R., Kothary N. (2017). The Role of Cone-Beam CT in Transcatheter Arterial Chemoembolization for Hepatocellular Carcinoma: A Systematic Review and Meta-analysis. J. Vasc. Interv. Radiol..

[60] Lucatelli P., De Rubeis G., Corradini L.G., Basilico F., Di Martino M., Lai Q., Corradini S.G., Cannavale A., Nardis P.G., Corona M. (2020). Intra-procedural dual phase cone beam computed tomography has a better diagnostic accuracy over pre-procedural MRI and MDCT in detection and characterization of HCC in cirrhotic patients undergoing TACE procedure. Eur. J. Radiol..

[61] Kim A.Y., Miller A. (2016). Evaluation of Surefire’s precision direct-to-tumor embolization device to augment therapeutic response to intra-arterial, liver-directed therapies for patients with primary and secondary liver cancers. Expert Rev. Med. Devices.

[62] Hoven A.F.V.D., Lam M.G.E.H., Jernigan S., Bosch M.A.A.J.V.D., Buckner G.D. (2015). Innovation in catheter design for intra-arterial liver cancer treatments results in favorable particle-fluid dynamics. J. Exp. Clin. Cancer Res..

[63] Pasciak A.S., McElmurray J.H., Bourgeois A.C., Heidel R.E., Bradley Y.C. (2015). The Impact of an Antireflux Catheter on Target Volume Particulate Distribution in Liver-Directed Embolotherapy: A Pilot Study. J. Vasc. Interv. Radiol..

[64] Petruzzi P., Crocetti L., Lencioni R. (2013). Chemoembolization of Hepatocellular Carcinoma. Semin. Interv. Radiol..

[65] Chung J.-J., Yu J.-S., Kim J.H., Kim K.W. (2010). Haemodynamic events and localised parenchymal changes following transcatheter arterial chemoembolisation for hepatic malignancy: Interpretation of imaging findings. Br. J. Radiol..

[66] Malagari K., Theodoros K., Maria P., Hippokratis M., Emmanouil E., Themis S., Vlassios S., Savvas T., Dimitrios K., Alexios K. (2016). Pharmacokinetics, Safety, and Efficacy of Chemoembolization with Doxorubicin-Loaded Tightly Calibrated Small Microspheres in Patients with Hepatocellular Carcinoma. CardioVasc. Interv. Radiol..

[67] De Baere T., Plotkin S., Yu R., Sutter A., Wu Y., Cruise G.M. (2016). An In Vitro Evaluation of Four Types of Drug-Eluting Microspheres Loaded with Doxorubicin. J. Vasc. Interv. Radiol..

[68] Lima M., Dutra S., Gomes F.V., Bilhim T., Coimbra E. (2018). Risk Factors for the Development of Postembolization Syndrome after Transarterial Chemoembolization for Hepatocellular Carcinoma Treatment. Acta Méd. Port..

[69] Gomes F.V., Oliveira J.A., Correia M.T., Costa N.V., Abrantes J., Torres D., Pereira P., Ferreira A.I., Luz J.H., Spaepen E. (2018). Chemoembolization of Hepatocellular Carcinoma with Drug-Eluting Polyethylene Glycol Embolic Agents: Single-Center Retrospective Analysis in 302 Patients. J. Vasc. Interv. Radiol..

[70] Mosconi C., Cappelli A., Pettinato C., Golfieri R. (2015). Radioembolization with Yttrium-90 microspheres in hepatocellular carcinoma: Role and perspectives. World J. Hepatol..

[71] Patel I.J., Rahim S., Davidson J.C., Hanks S.E., Tam A.L., Walker T.G., Wilkins L.R., Sarode R., Weinberg I. (2019). Society of Interventional Radiology Consensus Guidelines for the Periprocedural Management of Thrombotic and Bleeding Risk in Patients Undergoing Percutaneous Image-Guided Interventions—Part II: Recommendations. J. Vasc. Interv. Radiol..

[72] Chehab M.A., Thakor A.S., Tulin-Silver S., Connolly B.L., Cahill A.M., Ward T.J., Padia S.A., Kohi M.P., Midia M., Chaudry G. (2018). Adult and Pediatric Antibiotic Prophylaxis during Vascular and IR Procedures: A Society of Interventional Radiology Practice Parameter Update Endorsed by the Cardiovascular and Interventional Radiological Society of Europe and the Canadian Association for Interventional Radiology. J. Vasc. Interv. Radiol..

[73] Bilbao J.I., De Martino A., De Luis E., Díaz-Dorronsoro L., Alonso-Burgos A., De La Cuesta A.M., Sangro B., De Jalón J.A.G. (2009). Biocompatibility, Inflammatory Response, and Recannalization Characteristics of Nonradioactive Resin Microspheres: Histological Findings. Cardiovasc. Interv. Radiol..

[74] Memon K., Lewandowski R.J., Kulik L., Riaz A., Mulcahy M.F., Salem R. (2011). Radioembolization for Primary and Metastatic Liver Cancer. Semin. Radiat. Oncol..

